# Synchronization of Independent Neural Ensembles in Human EEG during Choice Tasks

**DOI:** 10.3390/bs9120132

**Published:** 2019-11-28

**Authors:** Alexander Zaleshin, Galina Merzhanova

**Affiliations:** Institute of Higher Nervous Activity and Neurophysiology, 5A Butlerova St., 117485 Moscow, Russia; merzhan@ihna.ru

**Keywords:** behavior, choice, risk, anxiety, impulsivity, EEG rhythms, time lag

## Abstract

During behavioral experiments, humans placed in a situation of having to choose between a more valuable but risky reward and a less valuable but guaranteed reward make their decisions in accordance with external situational factors and individual characteristics, such as inclination to risk or caution. In such situations, humans can be divided into “risk-inclined” and “risk-averse” (or “cautious”) subjects. In this work, characteristics of EEG rhythms, such as phase–phase relationships and time lags between rhythms, were studied in pairs of alpha–beta and theta–beta rhythms. Phase difference can also be expressed as a time lag. It has been suggested that statistically significant time lags between rhythms are due to the combined neural activity of anatomically separate, independent (in activation/inhibition processes) ensembles. The extents of synchronicity between rhythms were compared as percentages between risk-inclined and risk-averse subjects. The results showed that synchronicity in response to stimuli was more often observed in pairs of alpha–beta rhythms of risk-averse subjects compared with risk-inclined subjects during the choice of a more valuable but less probable reward. In addition, significant differences in the percentage ratio of alpha and beta rhythms were revealed between (i) cases of synchronization without long time lags and (ii) cases with long time lags between rhythms (from 0.08 to 0.1 s).

## 1. Introduction

Currently, the topics of learning and decision-making related to the probability of reward associated with risk are considered from different points of view and using different approaches. In a review paper, Rangel et al. [1] investigated different aspects of decision-making, goal-directed systems, risk, and uncertainty. The authors studied not only behavioral aspects of the problem, but also perception mechanisms operating during the decision-making process. A variety of studies have revealed the cortical and subcortical structures responsible for behavior connected to taking a risk when making a certain decision. Cohen and Ranganath [2] showed that the prefrontal cortex, corpus amygdaloideum, and ventral striatum play a considerable role in the decision-making process. During experiments, subjects chose between high-risk (i.e., with a low probability of large monetary reward) and low-risk (with a high probability of low reward) options. According to Donnelly et al. [3], the medial prefrontal cortex, ventral striatum, and nucleus accumbens could be the key areas in control of behavior for future reward, and dysfunction in these areas may cause increased impulsivity. Daw and Doya [4] showed the role of brain structures such as the striatum and the frontal and parietal cortex in choice based on risk of a probable reward. 

Many researchers, such as Drechsler et al. [5], Crowley et al. [6] have investigated decision-making and risk-taking behavior among adolescents and preadolescents who have suffered monetary losses. Drechsler et al. [5] presented two options: one with high gains and a major probability of losing and another with small gains and a high probability of winning.

A number of studies have investigated risky decision-making using EEG analysis. Massar et al. [7] studied EEG activity in adolescents during risky decision-making with various reward probabilities using the Iowa Gambling Task, and examined whether the relations were associated with differences in reward or punishment sensitivity. They replicated the relationship found earlier by showing a positive association between theta/beta ratio and risky decision-making. 

The new approach presented in our paper considered the relationship between the time lags in the phases of EEG rhythms and the characteristic features of behavior in decision-making tasks. The study of the phase–time synchronicity of EEG rhythms and features of time lags between rhythms can be useful for understanding the nature of risky decision-making (in particular, for understanding behavior in adolescence) and will serve as a basis for further works. 

Lachaux et al. [8] presented a method of direct quantitative evaluation of rhythm-specific synchronization (i.e., transition phase synchronization) between two signals. The authors discovered synchronization in the gamma rhythm (45 Hz) between isolated areas (e.g., hippocampus and coronal gyrus) and local synchronization in parts of the limbic area located within several centimeters of each other. Von Stein and Sarnthein [9] studied the synchronization of closely located areas of the temporal and parietal cortex within the beta rhythm, and interactions of isolated areas of the frontal and parietal cortex within the alpha and theta rhythms. In that study, the authors calculated phase synchronization between neurons of different cortex areas. Canolty et al. [10] presented a novel method of multivariable estimation of phase interaction as an addition to the method of phase–amplitude cross-frequency coupling, where a phase of a low-frequency signal modulates the range or power of a high-frequency signal as well as the range of rhythm frequency from delta to gamma. Cadieu and Koepsell [11] showed corticocortical interactions in the gamma range using phase coupling estimation. Another interesting study [12] revealed relationships between the low-frequency component of the signal and the amplitude envelope of the high-frequency component in various combinations, with a 40 ms correlational delay between envelopes and low-frequency components. 

Fixed-phase calculations of time events have been used by researchers in the analysis of rhythmic interactions. Researchers have demonstrated the use of fixed-phase calculations in the analysis of rhythmic interactions. Florin and Baillet [13] tested whether the identification of certain low-frequency events is essential at all. The authors considered both peaks and troughs of the low-frequency phase as phase events of interest. They showed that cortical occurrences of high-frequency oscillatory activity are conditioned to the phase of slower spontaneous fluctuations in neural ensembles. Cohen et al. [14] examined EEG theta, alpha, and beta frequency bands in the human medial frontal cortex while subjects played a competitive decision-making game. Phase–amplitude coupling during decision-making and the phase–amplitude coupling difference in various task conditions (e.g., loss and win) were investigated. Merzhanova et al. [15] showed that the moments of time in which the rhythm took a value equal to zero were determined, and the sinusoidal representation of the rhythm had a zero phase; such moments of time determine so-called zero markers.

Phase difference can also be expressed as a time lag. Based on this assumption, Merzhanova et al. [15] studied different types of phase rhythm interaction and showed that synchronicity in pairs of alpha–beta rhythms was increased in “risk-averse” (or “cautious”) people compared with “risk-inclined” people when choosing a more valuable but less probable reward. However, the synchronicity of theta and beta rhythms was identical for the subjects of both groups. The appearance of impulsivity and risk in EEG rhythms has been shown in a number of decision-making studies. Cavanagh et al. [16] showed that the beta rhythm decreased in the presence of risk, but the authors also cited references with the opposite results. De Pascalis et al. [17] showed that amplitude and phase were studied during synchronization in theta, beta, and gamma ranges. In a learning task with a monetary reward, the increase in the theta rhythm appeared for cases of increased impulsivity. In addition, impulsivity and risk are associated with a simultaneous decrease in both alpha and beta rhythms [18]. 

The aim of this work was to evaluate the characteristics of EEG rhythm pairs based on the parameter of phase synchronicity (with or without a fixed time correction for all zero markers of the second rhythm in any pair) in subjects in a situation of choosing between the probability and value of the reward in order to identify the individual specifics of “riskiness/caution”. In addition, we sought to study not only cases with synchronicity but also cases with time lags, which increased the possibility of analyzing frequency relationships of closely spaced frequencies.

## 2. Materials and Methods

Subjects: A total of 33 healthy subjects (aged 24 ± 4 years, 15 male/18 female) took part in the study. These participants, students and trainees, had previously received information about our study and were interested in the nature of risky behavior. They confirmed the absence of color blindness and neurological or mental disorders. All participants were right-handed. Before the study, every subject gave written consent for participation in research. This study was approved by the Ethics Committee of the Institute of Higher Nervous Activity and Neurophysiology.

Stimuli: The experiments involved an expectation of reward when choosing an object on the left or right side of a screen (Cohen et al. [19] used the same approach). On a 17 inch monitor screen that was placed 50 cm from the subject, images of two gray circles (12 cm in diameter) appeared on a dark-gray background. Stimuli for a yes/no decision were represented by green and red circles. Colored circles then alternated with the two gray circles; all circles were of the same size and location. All circles were located on a horizontal plane, with the green stimulus on the left and the red stimulus on the right.

After the two circles appeared, the subject was presented with alternatives: to choose the green circle and be awarded one point with a probability of 100%, or the red circle and be awarded six points with one of a range of fixed probabilities (0%, 10%, 25%, 50%, 75%, or 100%). Each subject took part in six experiments, with 0%, 10%, 25%, 50%, 75%, and 100% probabilities of receiving six points upon choosing the red circle. The choices were proposed in 200 trials for each experiment. The probability of receiving six points when selecting the red circle was unknown to the subject during each experiment. The task of the subject in every experiment was to get the largest number of points, which earned him or her an additional reward.

**Figure 1:**
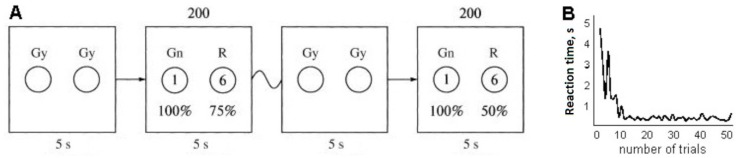
Diagram showing the images presented to the subject on the monitor screen. Gray circles (Gy) show the beginning of the series of choices or a change in the probability of the reward; green circles (Gn) and red circles (R) show the alternative choices. The time of presentation to the subject was 5 s for each pair of circles. The probability of receiving one point on selection of the green circle (Gn) was always 100%; the probability of receiving six points on selection of the red circle (R) ranged from 0% to 100% in each experiment and was unknown to the subject. The number of trials was 200 in one experiment (**A**). Typical reaction time of one experiment (**B**).

Thus, the red circle yielded more points but the probability of receiving them changed in different series of experiments (Figure 1A). The choice was made using the left mouse button for the green circle and the right button for the red circle. In each trial, the presentation on the monitor screen for the participant lasted until the color circle was selected, but not more than 5 s. 

The mean time for selecting one color circle in the first 10 trials of one experiment by each participant varied considerably. The mean time for selecting one color circle in the subsequent 190 trials of one experiment was about 470 ms (Figure 1B). The difference in the speed of choosing a red or green circle for the purposes of our experiment was not a priority (Blizzard et al. [20] found that the mean reaction time to a red stop signal was approximately 25 ms faster than that to a green stop signal).

EEG: During each experiment, the subject’s EEG was recorded. During the EEG recoding, the subject sat in a chair in a darkened, relatively soundproofed, and screened chamber. Monopolar EEG traces were measured from 16 leads in relation to ear electrodes: Fp1, Fp2, F3, F4, F7, F8, C3, C4, O1, O2, T3, T4, T5, T6, P3, and P4. To remove power-line noise, a 50 Hz notch filter was applied during EEG recordings.

After preamplification and amplification using a 16 channel amplifier from MBN (Moscow, Russia), EEG traces were digitized with a bandpass of 70 Hz and a sampling frequency of 1 kHz, and were recorded in computer memory for subsequent processing. The bandwidth was filtered to maintain a frequency range higher than 1 Hz. Segments with motor artifacts were excluded from analysis. Analysis was based on EEG segments, which included traces from 1 s before and 1 s after the stimulus was displayed.

In the experiments, rhythm records were filtered; if they differed by less than 10 ms in two channels or more, taking the time lag into account, they were excluded from the analysis in every channel. 

Maex and De Schutter [21] considered the issues of time lags in synchronization According to that paper, brain rhythms appear due to synchronization of neurons and their tuning to an activity pattern, and networks of mutually related inhibitory neurons are involved in this process. Bush and Sejnowski [22] studied synchronization of neurons in the neocortex and the time lag in synchronization; their study took into account the mutual inhibition of inhibitory neurons in one column, as well as synchronization of neurons between columns.

Statistical analysis: The experiments compared revealed rhythms (and pairs of rhythms) and behavior at the same moment in time and the difference between recorded rhythms (and pairs of rhythms) at different moments in time, including constant time shifts relative either to the stimulus or to other markers. Rhythms that occurred close to the instant at which the stimulus switched on were detected, as well as rhythms with a long time lag (several hundred milliseconds). We searched for rhythms that occurred with the same time lag before or after the stimulus but not concurrently. 

A fourth-order Butterworth filter was used to resolve each of the 16 traces from each experiment into the following ranges: delta (1–4 Hz), theta (4–8 Hz), alpha (8–13 Hz), and beta (13–30 Hz). Subsequently, time values of the initial phase of each sine wave for all band rhythms (zero markers) were determined. For further analysis, Spike 2 software was used for building interval, poststimulus (PSHs), and cross-correlation histograms (CCHs). The interval histograms were built for alpha–beta pairs according to time intervals between zero markers in alpha and beta rhythms. Sreenivas and Niederjohn [23] applied a similar method: in their work the statistical properties of intervals between moments of zero-crossing were used in studying a signal damaged by noise. 

Here, we assumed that a high temporal variation between rhythms was partially offset by additional screening of interelectrode temporal fluctuations. To compensate for remote effects, the following method was used: phase lags in rhythm pairs were analyzed for each electrode lead over separate segments of the cortex, and only after that were the contributions of all EEG leads summarized. 

The induced responses of rhythms of each range to the presented stimuli were analyzed for zero markers using the PSHs, CCHs, and interval histograms. Triggers in the CCHs were rhythms of alpha, beta, and theta rhythms, alternately. A main trigger in the PSHs (and an additional trigger in the CCHs and interval histograms) was the moment of appearance of red and green circles; analysis included records with the red circle set at various reward expectations. Using zero markers, the PSHs, CCHs, and interval histograms were built with an analysis ranging from −1 s before stimulus to +1 s after stimulus. A bin of 33.3 ms was used in PSHs, and a bin of 5 ms was used in CCHs and interval histograms. For each probability of reward for each subject, 64 histograms with 16 leads and 4 rhythms were analyzed. The CCHs were built according to rhythms, divided by ranges, to identify the synchronicity of their pairwise occurrences. Thus, the constructed CCHs referred to correlations between alpha–beta, alpha–theta, and beta–theta rhythms. For each probability and for each subject, we considered 6, 18, and 48 histograms with 2, 6, and 16 leads, respectively. The arithmetic average was calculated using 2, 6, and 16 CCHs for each combination of three rhythms for each probability, and the average value was also calculated for each histogram after summation.

Peaks exceeding the mean by three sigma were identified in relation to the number of histograms analyzed. Significant peaks on the CCHs are evidence of a high degree of correlation between rhythms of the pair of ranges under analysis. The extents of phase synchronicity between rhythms (with or without a fixed time correction for all zero markers of the second rhythm in any pair), as percentages, were compared in risk-inclined and risk-averse subjects using Fisher’s test. 

## 3. Results

The behavior of the subjects in the experiments can be divided into two stages: the first 10 choice trials, in which the individual under study showed the most expressive inclination to risk, and the remaining 190 trials, in which individual parameters of learning the algorithm to gain the maximum number of points, for a given probability of obtaining six points on selecting the red circle, became apparent.

During experiments with a probabilistic receipt of a valuable reward (0%, 10%, 25%, 50%, 75%, and 100%), subjects showed stable behavioral strategies. Based on the selected behavioral strategy, all subjects were classified into two groups (risk-inclined and risk-averse) according to their preferences for choosing red and green circles during all 200 trials. Subjects’ questionnaires showed that during the experiment, they tended to receive maximal numbers of points but did not choose any suitable fixed algorithm that would seem to be “optimal”.

The differences in the total number of collected points between groups of risk-inclined and risk-averse subjects were maximal at the 25% probability of receiving the valuable reward. This is due to the fact that with a probability of 25%, participants received about the same winnings regardless of the strategy of behavior and scored about the same number of points. As a result, participants were not able to consciously calculate the best way to get the maximum number of points, and the behavior of the subjects was determined only by their inclination to risk.

**Figure 2:**
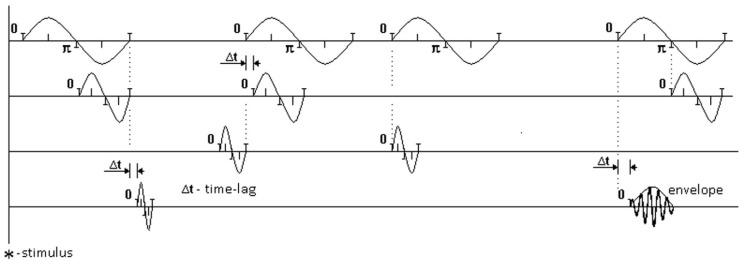
Illustration of sets of EEG rhythms. Different variants of time lags between moments of zero-crossing are presented. * indicates the moment when the stimulus was presented.

Figure 2 illustrates sets of EEG rhythms. Each oscillation schematically shows the record of one distinct rhythm at one moment of zero-crossing. The moments of zero-crossing in one rhythm are compared with the moments of zero-crossing in another rhythm. Times for comparing pairs of rhythms are shown in dotted lines for distinct oscillations. 

**Figure 3:**
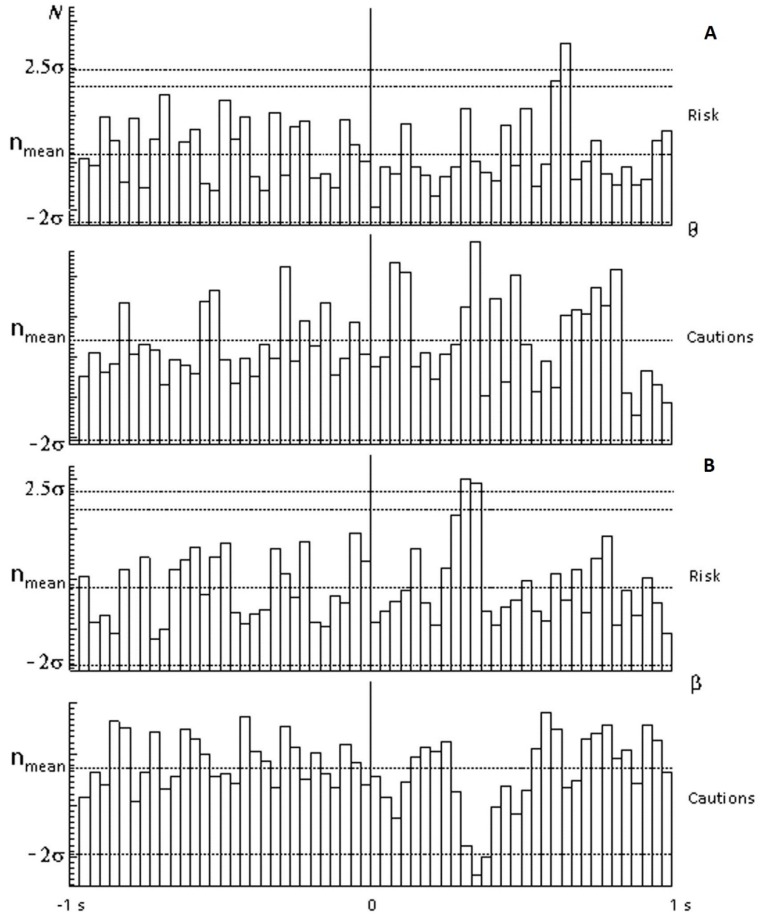
Differences in appearance of the theta and beta EEG rhythms in response to stimuli on selection of the red circle with a 25% probability of receiving the valuable reward for members of the “risk-inclined” and “risk-averse” (or “cautious”) groups. On the poststimulus histogram (PSH), the lower and upper two horizontal lines show ±2σ and 2.5σ, respectively, in relation to the histogram mean. The horizontal axis shows time in seconds; the vertical axis shows the number of times the marker crossed the null phase of each rhythm (N). Members of the risk-inclined group (Risk) were seen to be characterized by activation of the theta and beta rhythms, while members of the cautious group (Cautious) were characterized by inhibition of the beta rhythm.

Figure 3 shows spectral (for beta ranges and for theta ranges) and temporal (in response to selection of red circles) EEG responses typical of members of the risk-inclined (above) and cautious (below) groups under conditions of a 25% probability of receiving the valuable reward. The theta range (Figure 3A) showed a significant peak (2.5 sigma from mean) on selection of the red circle by risk-inclined subjects, indicating the appearance of a rhythm in the theta range with a latency of 600 ms, in contrast to the cautious group, in which this rhythm was not marked. In terms of rhythms in the beta range (Figure 3B), risk-inclined subjects showed a significant peak with a latency of 300 ms, in contrast to cautious subjects, in which there was inhibition of this rhythm with essentially the same latency. In terms of delta and alpha ranges, there were no significant differences between cautious and risk-inclined subjects.

**Figure 4:**
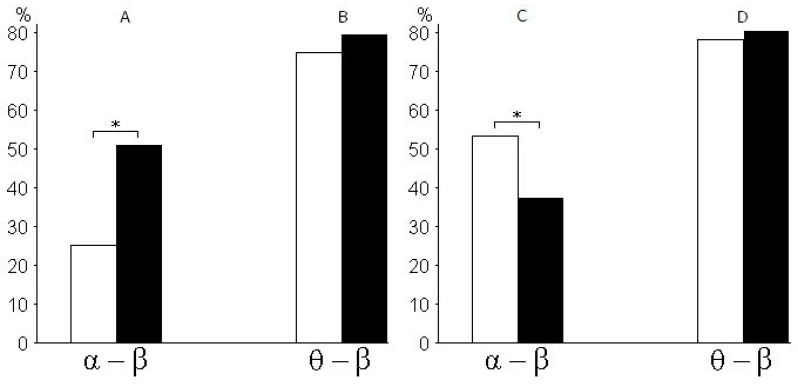
Percentage ratio in synchronization between pairs of EEG rhythms. The horizontal axis shows variants of pairs of rhythms—(alpha and beta) and (theta and beta)—for both groups. The vertical axis shows the percentage ratio in pairs of rhythms (%) for the cases of synchronicity (**A**,**B**) and time lag (from 0.08 to 0.1 s) between rhythms (**C**,**D**). Percentage ratios for risk-inclined subjects are shown in white columns and for risk-averse subjects in black columns for all cases (A–D). * *p* < 0.05 was considered a statistically significant difference.

Figure 4 shows the percentage ratio in synchronization between pairs of alpha and beta rhythms of EEG (Figure 4A), the percentage ratio in synchronization between pairs of theta and beta rhythms (Figure 4B), the percentage ratio in pairs of alpha and beta rhythms with a time lag (from 0.08 to 0.1 s) between rhythms (Figure 4C), and the percentage ratio in pairs of theta and beta rhythms with a time lag (from 0.08 to 0.1 s) between rhythms (Figure 4D) with a 25% probability of receiving the valuable reward for subjects of risk-inclined and risk-averse groups. According to the histograms, risk-inclined subjects showed more alpha–beta pairs with a large variation between alpha and beta, but fewer alpha–beta pairs with small variations. That is, in risk-inclined subjects, small variations between alpha and beta rhythms occurred in only a small number of alpha–beta pairs. The opposite results were found in the group of risk-averse subjects.

**Figure 5:**
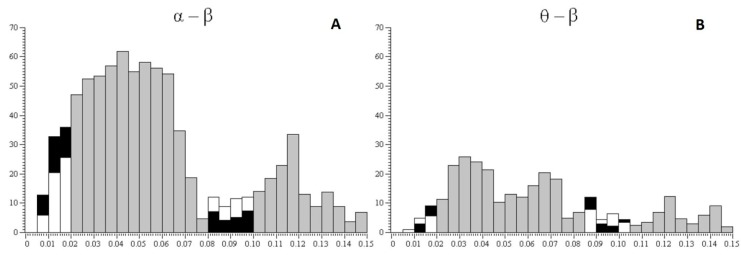
Averaged interval histograms between alpha and beta rhythms and between theta and beta rhythms. The abscissa shows time intervals between pairs of rhythms, alpha and beta and theta and beta, in seconds; the ordinate shows the number of intervals; white columns show the risk-inclined group and black columns show the risk-averse group. Gray columns indicate ranges for which the calculation of cross-correlation histograms (CCHs) was not performed; in this case, average data are presented for all subjects.

Averaged interval histograms between alpha and beta rhythms (Figure 5A) and between theta and beta rhythms (Figure 5B) for a 25% probability of receiving the valuable reward shown for subjects of both groups. 

## 4. Discussion

This work showed that the processes underlying decision-making associated with risk, or with the receipt of rewards, are reflected not only in behavioral reactions but also in EEG activity. This approach to studying risk behavior with EEG may shed additional light on the neurocognitive processes underlying risk judgments.

The main hypothesis of this work was that (i) different types of EEG rhythms in humans do not usually occur simultaneously in response to an external stimulus, and (ii) the sequence of occurrence of pairs of rhythms in cautious people differs from the sequence of occurrence of pairs of rhythms in risky people. In this paper, it was shown that when subjects with different behavior tendencies were presented with a stimulus, the alpha and beta rhythms followed each other in significantly different combinations, which, in particular, was observed in the time lags between rhythms.

Händel and Haarmeier [24] analyzed the phase components of different EEG rhythms and examined the idea that the relationship between amplitude and phase between low- and high-frequency brain oscillations makes it possible to detect EEG responses to a stimulus and also to identify weak high-frequency signals. These authors [24] showed that amplitude modulation of occipital high-frequency oscillations in a range of 63 ± 5 Hz occurs in the slow-frequency phase (1–5 Hz). They expressed the view that a correlation of high- and low-frequency rhythms in response to a visual stimulus takes place when detecting the visual stimulus. Our study expanded and modified the methods of phase–amplitude analysis in relation to pairs of low-frequency rhythms, and it could be further applied to the study of clinical disorders. 

The interaction of rhythms can be considered in terms of the interaction of neural ensembles. Independent neural ensembles can have rare and short-term synchronization; delayed synchronization with a large temporal spread is also possible. At the same time, several independent ensembles may be active in a short time interval.

Redish et al. [25] suggested that neural ensembles for hippocampal pyramidal cells are independent in activity correlation and closely spaced. As one of the variants for synchronizing independent ensembles, externally determined synchronization can be considered, which occurs in response to stimuli of the same or different modality. For example, Yu et al. [26], and van Diepen et al. [27] studied parallel tactile, visual, and auditory stimuli. Loss of dopamine in cases of Parkinson’s disease led to a decrease in oscillation frequency and an increase in synchronization activity [28]. Based on the cross-correlation of neuronal activity, neural pairs with uncorrelated activity or activity with a variable phase lag involving globus pallidus neurons can act as independent oscillators.

Phase synchronicity as a possible mechanism for integration of brain processes has been the topic of many studies. Varela et al. [29] showed that neural ensembles were described as distributed interconnected networks of neurons. König et al. [30] showed that the occurrence of synchronicity at remote distances in the visual cortex of a cat is associated with oscillatory activity in the respective groups of cells. According to the authors, their samples of oscillations associated with synchronization at a remote distance always showed frequencies in the gamma rhythm. Koutsoukos et al. [31] analyzed spontaneous synchronization and studied interfrequency interactions using phases of rhythms. The authors suggested that synchrony in brain activity is a mode that reflects the collective behavior of neural ensembles. Canolty et al. [32] studied the amplitude, beta rhythm phase, and spike activity of neuron ensembles; their study showed that the spike activity of individual neurons is coupled with the motor beta rhythm (10–45 Hz) by several parameters. Fries [33] suggested that synchronization affects the communication between neuronal groups. Fast modulation of excitation of a neural group with synchronization in the gamma rhythm (30–90 Hz) allows avoidance of subsequent inhibition and effectively activates postsynaptic neurons. The author studied the spike probability dependent on the gamma rhythm phase function. In addition, the author suggested the presence of a “bottom-up” hierarchic influence in rhythms, for example, influence of alpha and beta rhythms on the gamma rhythm pattern. As a result, several interacting rhythms set effective, precise, and selective interactions among neurons. Fries [34] presented the idea that an effective communication structure is realized under the condition of the existence of coherence patterns between groups of neurons, and is usual for phase inhibition in neural oscillations. The authors of the abovementioned papers consider various characteristics of neural ensembles to be applicable both to brain rhythms and separate neurons. 

Gollo et al. [35] studied so-called phase and antiphase synchronizations with zero lag for spatially separated areas of the cortex. Barardi et al. [36] studied the interaction time between populations of neurons in different brain areas located close to or remote from each other. Vicente et al. [37] investigated how, despite temporal delays, the reciprocal interactions between two brain regions can lead to the associated neural populations to fire in unison. The authors suggested that the zero-time-lag synchrony among such distant neuronal ensembles must be established by mechanisms that are able to compensate for the delays involved in neuronal communication. The results indicated the presence of zero-lag synchronization while taking into account that the time lag of interaction between close neurons is a few milliseconds, while between different areas, it may be tens of milliseconds. In addition, Vicente et al. [37] claimed that the presence of synchronization with zero lag allows for maximum communication. 

Gollo et al. [38] studied timing with a phase lag in the theta rhythm between the front and visual cortex to determine whether the theta oscillation in the hippocampus is responsible for synchronization between these areas. Several mechanisms of synchronization between different structures of the cortex, particularly mutual inhibition and mutual excitation of neuronal populations, were shown by Chawla et al. [39]. For areas located on different levels of the visual hierarchy, systematic local phase lags occurred. The authors’ calculations showed that for groups of three, zero-lag synchronicity occurred more frequently than for pairs of interacting areas. In terms of electrophysiological data, functional interactions are often defined by consistency of phase angles between two or more electrodes located in the same or different areas of the brain [40]. Lachaux et al. [41] examined gamma activity in response to visual stimuli. Using a phase matrix of signals from electrodes, it was shown that evoked potentials on most electrodes can be the same as if there is a time delay (100 ms). The authors of these papers do not share a single point of view on how the compensatory coordinated lag defined by the difference in signal paths, which is intended to establish zero-lag synchrony between remote areas of the brain, including those receiving signals of different modalities, occurs.

Eckhorn et al. [42] explained how visual objects are presented by synchronization in the visual cortex. The local synchronicity by the gamma activity phase in the primary visual cortex was examined within several millimeters. The authors posited that the hypothesis of association processes via synchronization should be expanded to a general time coding across the entire frequency spectrum of cortex activity, with phase and amplitude interactions among transient and stochastic signals. Rudrauf et al. [43] described an approach to analyzing synchronization dynamics making it possible to trace characteristics of phase synchronicity between groups of signals. Cohen [44] suggested modification of standard methods of analysis for calculating the time of the instantaneous frequency series based on the time derivative of the time series phase angle. Hafting et al. [45] argued that the precession phase at the output of the hippocampus (such as the prefrontal cortex) may indicate that the activity recorded occurs in the prefrontal cortex, and other output structures (a) inherit the phase precession from the hippocampus or (b) express this activity independently of the hippocampus. The authors of the abovementioned papers analyzed phase characteristics and phase synchronicity.

Naruse et al. [46] and Takeichi et al. [47] analyzed induced activity of the alpha rhythm as a reaction to the presentation of a verbal pattern when the EEG rhythm was used as the evaluation parameter for amplitude and phase balance. More detailed work could include an analysis of pulsed bursts occurring on the rising and falling edges of a sinusoid signal, which is widely used in various scientific tasks [48,49]. Applied to our topic, the method for EEG evaluation using markers of fixed values of EEG rhythm phases followed by the construction of a CCH [24] is the most suitable one.

When studying financial reward tasks, it has been found that some subjects (risk-averse) prefer to receive less money for certain rather than a greater amount in a smaller percentage of cases, while for others subjects (who are not afraid of a high level of risk), the opposite is true [50,51]. While the first form of choice is often referred to as behavioral and the second one as cognitive, Kahneman and Tversky [51], as well as Rachlin et al. [50], have suggested that both types of choices involve cognitive processes. 

Risk-taking is a complex process that involves evaluation of the reward and the risk level. It was shown that risk-taking subjects achieved a better final result. On the other hand, we cannot ignore the fact that during the experiments, some risk-inclined subjects managed to determine the probability in a specific experiment and calculated the risk benefit. 

## 5. Conclusions

1. During experiments, participants were placed in a situation of having to choose between a more valuable but risky and a less valuable but guaranteed reward, with different probabilities of receiving high scores (0%, 10%, 25%, 50%, 75%, and 100%). Based on their behavioral strategies, the participants were divided into risk-inclined and risk-averse groups.

2. The differences in the total number of collected points between groups of risk-inclined and risk-averse subjects were maximal at the 25% probability of receiving the valuable reward. At this probability, participants were not able to consciously calculate the best way to get the maximum number of points, and the behavior of the subjects was determined only by their inclination to risk.

3. Significant synchronicity in pairs of alpha–beta rhythms was more often observed in the risk-averse group compared with the risk-inclined subjects when choosing the more valuable but less probable reward.

4. Alternatively, long time lags between rhythms (from 0.08 to 0.1 s) were more often observed in the risk-inclined group compared with the risk-averse subjects when choosing the more valuable but less probable reward.

5. Significant differences in the percentage ratio of pairs in the task of decision-making were not revealed between other pairs of rhythms (beta and theta, etc.)
